# Spatio-temporal modelling of malaria mortality in India from 2004 to 2013 from the Million Death Study

**DOI:** 10.1186/s12936-022-04112-x

**Published:** 2022-03-17

**Authors:** Sayantee Jana, Sze Hang Fu, Hellen Gelband, Patrick Brown, Prabhat Jha

**Affiliations:** 1 Indian Institute of Technology, Hyderabad, India; 2 Indian Institute of Management, Nagpur, India; 3 Dalla Lana School of Public Health, Centre for Global Health Research, St. Michael’s Hospital, University of Toronto, Toronto, ON Canada; 4 Department of Statistical Sciences, University of Toronto, Toronto, Canada; 5 Dalla Lana School of Public Health, University of Toronto, Toronto, Canada

**Keywords:** Spatio-temporal modelling, India, Million Death Study, Malaria mortality

## Abstract

**Background:**

India has a substantial burden of malaria, concentrated in specific areas and population groups. Spatio-temporal modelling of deaths due to malaria in India is a critical tool for identifying high-risk groups for effective resource allocation and disease control policy-making, and subsequently for the country’s progress towards United Nations 2030 Sustainable Development Goals.

**Methods:**

In this study, a spatio-temporal model with the objective of understanding the spatial distribution of malaria mortality rates and the rate of temporal decline, across the country, has been constructed. A spatio-temporal “random slope” model was used, with malaria risk depending on a spatial relative risk surface and a linear time effect with a spatially-varying coefficient. The models were adjusted for urban/rural status (residence of the deceased) and Normalized Difference Vegetation Index (NDVI), using 2004–13 data from the Million Death Study (MDS) (the most recent data available), with nationwide geographic coverage. Previous studies based on MDS had focused only on aggregated analyses.

**Results:**

The rural population had twice the risk of death due to malaria compared to the urban population. Malaria mortality in some of the highest-risk regions, namely the states of Odisha and Jharkhand, are declining faster than other areas; however, the rate of decline was not uniformly correlated with the level of risk. The overall decline was faster after 2010.

**Conclusion:**

The results suggest a need for increased attention in high-risk rural populations, which already face challenges like inadequate infrastructure, inaccessibility to health care facilities, awareness, and education around malaria mortality and prevalence. It also points to the urgent need to restart the MDS to document changes since 2013, to develop appropriate malaria control measures.

**Supplementary Information:**

The online version contains supplementary material available at 10.1186/s12936-022-04112-x.

## Background

Malaria contributes not only to the global infectious disease burden, but has serious economic consequences borne largely by those in their financially productive ages. Verbal autopsy studies in India report a national estimate of 130,000 malaria deaths before 70 years of age in 2015 [1], down from about 200,000 deaths at these ages in 2005 [2]. Malaria, like most other vector-borne diseases, is characterized by spatial and temporal variations due to climatic, ecological, and human factors. These variables can help predict spatio-temporal patterns of the disease and identify hot-spots to enable efficient disease monitoring, cost-effective allocation of resources, and ultimately, effective disease control [3–7].

Environmental factors are not solely determinative of the spatio-temporal distribution of vector-borne diseases. Political and state borders also play a major role, as they determine spatial distribution and implementation of control and prevention programmes. This is illustrated by a spatio-temporal study in Northern Thailand, where malaria incidence patterns contrast sharply with bordering Myanmar [5]. Furthermore, it has been argued that local disease variations cannot be accounted for by environmental or biological indicators alone [4].

Much of the current literature on spatio-temporal variation in risk of diseases uses ecologically-defined risk factors, i.e., average income by census area or satellite-based land use categories, to explain disease dynamics [7–10]. An advantage of this type of research is that data on spatio-temporal risk factors may be publicly available without the need for new data collection [3, 8]. Moreover, spatial analyses can illuminate correlations of geographical areas and small-area variations [11].

A few studies have investigated spatial and spatio-temporal distributions of malaria in specific states or districts of India [3, 12–17]. However, no other study seems to have explored spatio-temporal distribution across the entire country using actual mortality data for over a decade.

In this study, spatio-temporal models for malaria mortality in India, have been constructed, to investigate temporal trends and spatial distribution, with the aim of identifying the more vulnerable populations and regions. The rate of change in malaria mortality in high- and low-burden areas was also assessed. Particular emphasis has been put on the high-burden states, namely, Odisha, Jharkhand, Chhattisgarh, Madhya Pradesh and the North-eastern states, in the exploratory analysis.

## Methods

### Data sources

This study uses data from India's largest mortality survey, the Million Death Study (MDS), where data were collected by the Registrar General of India’s (RGI’s) Sample Registration System (SRS) in collaboration with the University of Toronto’s Centre for Global Health Research (CGHR) for the years 1998 to 2017. The RGI divides the country into 1 million small areas based on the 10-year censuses of 1991, 2001, and 2011, respectively, for the three different sampling frames [18]. About 8,000 of these small areas are randomly selected by the SRS and all births and deaths are monitored in about 1.3 million households in each round. The SRS sampling units are densely spread across the country (Additional file 1: Appendix A.2). Deaths in urban areas were geocoded to postal codes and deaths in the rural areas were geocoded to village locations. Because of greater inaccuracies in assigning cause of death (COD) in older adults [18–22], in this study, the focus is on deaths of people under 70 years of age. Results for older ages are provided in Table 1. Deaths with ICD-10 [23] codes B50-54 were assigned as death due to malaria. The data was collected during 2004–2013 by MDS, which had 7597 sampling units, of which 7416 were geocoded. Of these 7377 geocoded sampling units from the mainland, after excluding island units, were included. More recent data, that is, data beyond 2013 from MDS is not available. The MDS data were collected twice a year through verbal autopsies (VAs). Respondents from each household with a death in the last six months were interviewed by one of about 900 non-medical trained staff to gather information on the circumstances, symptoms, and treatments before death of household members, including a half-page local language narrative. These paper records were converted to electronic form and randomly assigned independently to two of 400 specially trained physician coders to assign the cause of death according to ICD-10, with differences undergoing anonymous reconciliation, and persisting differences adjudicated by a senior physician [24]. The VA uses a modified version of the WHO 2012 VA instrument which yields similar results to the longer WHO form [19]. United Nations (UN) population figures for India were used to calculate the age- and sex- specific death rates by applying the proportion of malaria deaths (weighted for sampling design) to the UN death totals in each year. Sub-national estimates of deaths used the relative SRS death rates applied to these UN totals.

**Table 1:** Malaria-attributed deaths from MDS (2004–13) by age-groups

Age Group	Deaths attributed to malaria	All coded deaths	Proportion of malaria deaths out of all deaths	Died in a health facility	Rural
2004–2006					
1–59 months	585	11,480	5.1	91	523
5–14 years	382	4311	8.9	77	338
15–29 years	377	8939	4.2	129	314
30–44 years	305	11,728	2.6	87	250
45–59 years	422	18,684	2.3	71	344
60–69 years	500	21,788	2.3	46	430
> 70 years	730	40,783	1.8	46	627
2007–2010					
1–59 months	656	12,600	5.2	146	593
5–14 years	376	4573	8.2	107	314
15–29 years	507	13,052	3.9	167	412
30–44 years	398	17,838	2.2	118	313
45–59 years	586	29,266	2	106	484
60–69 years	644	33,115	1.9	77	559
> 70 years	1041	62,028	1.7	72	893
2010–2013					
1–59 months	282	5909	4.8	74	246
5–14 years	173	2469	7	62	156
15–29 years	296	8976	3.3	110	244
30–44 years	296	12,586	2.4	78	241
45–59 years	403	22,439	1.8	94	328
60–69 years	427	25,482	1.7	64	363
> 70 years	701	50,074	1.4	49	580

### Spatio-temporal modelling

Generalized Linear Geostatistical Models (GLGMs), proposed by Diggle, Moyeed, and Tawn [25], were used to model non-Gaussian spatial data [26]. The number of deaths in each sampling unit and each year, were modelled with a Poisson distribution with the mean being the product of an age-adjusted expected count and a spatially- and temporally-varying relative risk. The relative risk contains an urban–rural effect, a non-parametric time trend for years, an effect for Normalized Difference Vegetation Index (NDVI), a spatially-varying risk surface, which is a spatially-varying random effect, meant to account for unobserved spatial risk factors, and a linear time trend with a spatially varying slope, also a random effect. It is this final term which captures spatio-temporal variation: a location having a value of zero for this second spatial surface is following the national time trend, and regions with negative or positive values for this surface have malaria rates declining more rapidly or more slowly, respectively, than the national average. The two spatial surfaces have Matérn correlation functions, where the correlation between two locations is a decreasing function of the distance between the locations. A spatially-independent unit-level random effect was also included to account for possible overdispersion. Bayesian inference via Integrated Nested Laplace Approximation (or INLA) [27] was used for model fitting. Besides being relatively simple, INLA models are accurate, very fast to run, and their model diagnostics and predictive measures are simple to implement [28]. Details of the model are provided in Additional file 1: Appendix A.1.

The correlation between the spatial relative risk and the spatially-varying random time trend quantifies the relationship between malaria prevalence at baseline and changes in prevalence over time. Were it the case that the highest-risk areas are catching up to the rest of India and improving more quickly, the correlation would be negative as high spatial relative risk would coincide with negative values of the time trend. The posterior correlation between the two spatial random effects was calculated by generating 700 posterior samples and computing the empirical correlation between the two spatial effects.

## Results

**Fig. 1:**
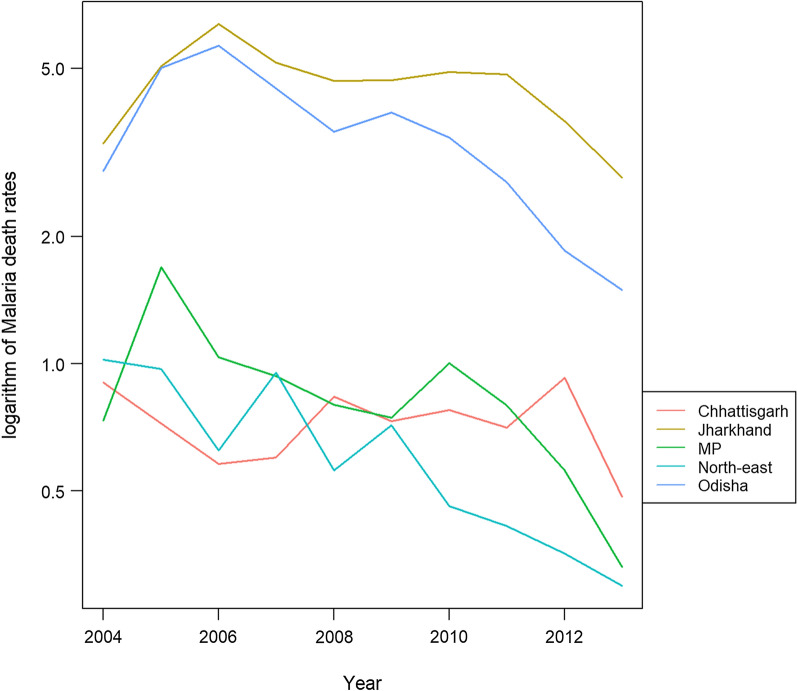
Raw annual malaria death rates, using MDS data (2004–2013) for high-burden states. *MP* Madhya Pradesh

Annual malaria death rates from the MDS decreased over the 2004–13 period, with the pattern of decrease varying across different high-burden states (Fig. 1). Table 1 presents the national figures for the proportion of malaria deaths, and of those how many were health-facility deaths and rural deaths, by age-group. Similar tables for the high-burden states have been included in Additional file 1: Appendix A.4.

**Fig. 2:**
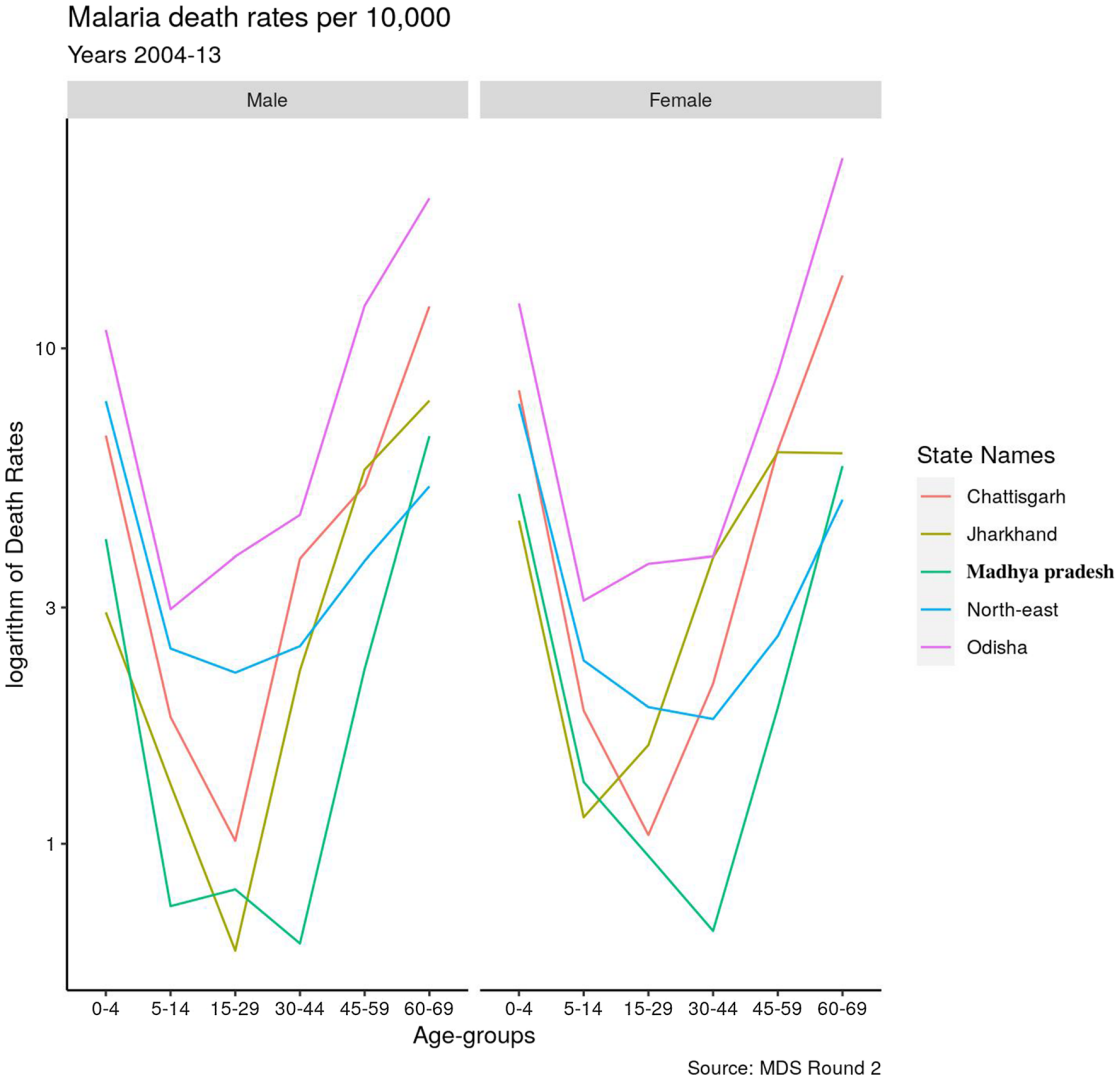
Age distribution of malaria mortality rates in rural areas across the high-burden states, for 2004–2013. *MP* Madhya Pradesh

A surprising finding by Dhingra et al. [2] using the MDS data was the U-shaped age-specific mortality pattern for malaria, with mortality rising at older age groups, contrary to earlier assumptions that malaria mortality is highest in young children and remains low throughout the rest of life in high-transmission areas. A similar U-shaped pattern for malaria mortality in the individual high-burden states, was observed, both in urban and rural stratifications (Fig. 2), and for males and females. Rural populations are at twice the risk of urban populations across all five high-burden states (Additional file 1: Appendix), which we explore further in the spatio-temporal models. Among the high-burden states, Odisha is uniformly highest across age groups and also across urban and rural stratifications. The North-eastern states are second behind Odisha for younger age groups but comparable to other states above age 44. Females are at a slightly higher risk than males.

**Fig. 3:**
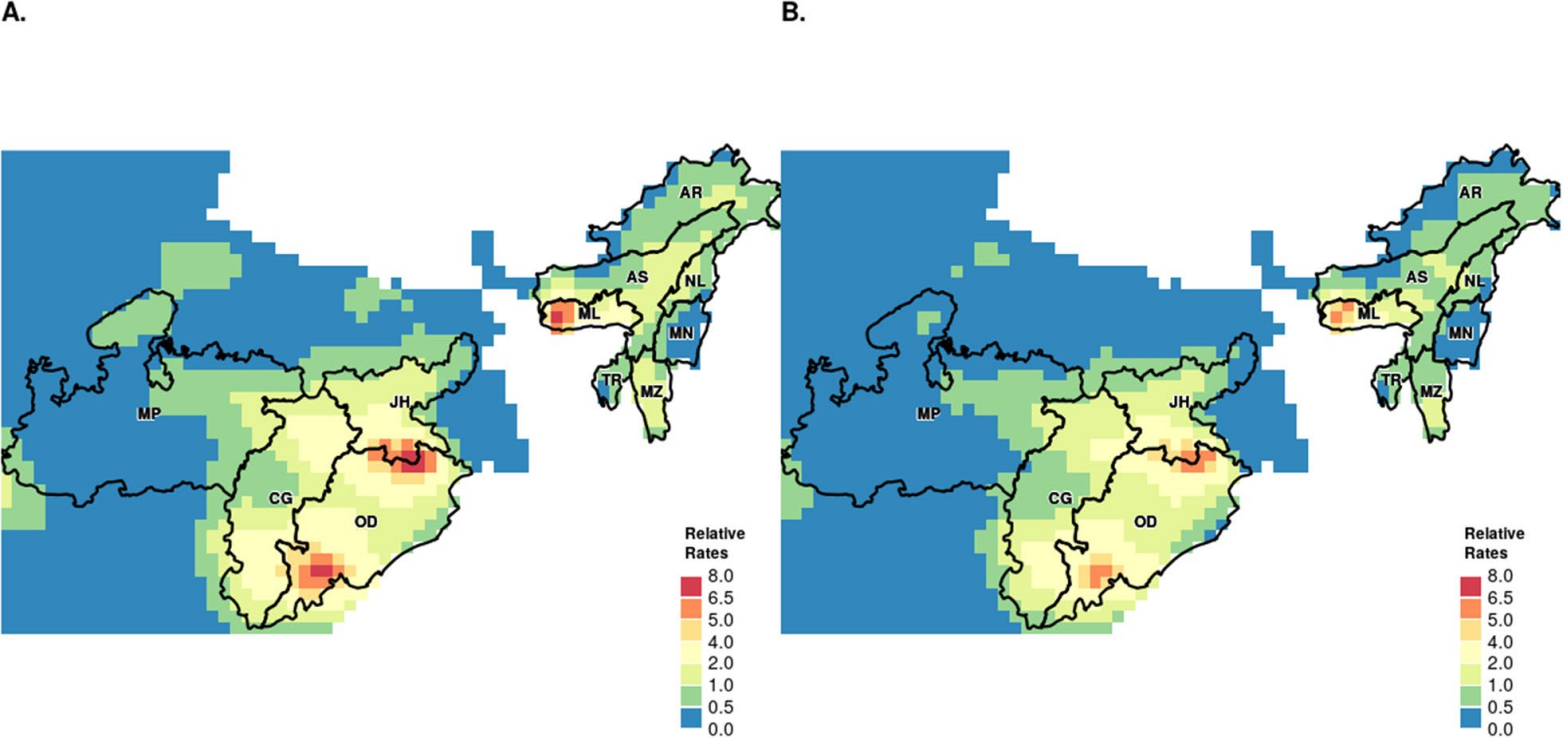
Predicted mortality rate. **A.** Age-adjusted malaria predicted mortality rate, relative to the national average for 2004. **B.** Age-adjusted malaria predicted mortality rate, relative to the national average for 2013. The black boundaries have been used to highlight the high-burden states. (State abbreviations: *AR* 'Arunachal Pradesh', *NL* 'Nagaland', *MN* 'Manipur', *MZ* 'Mizoram', *TR* 'Tripura', *ML* 'Meghalaya', *AS* 'Assam', *JH* 'Jharkhand', *OD* 'Odisha', *CH* 'Chhattisgarh', *MP* 'Madhya Pradesh')

The predicted spatial maps for the high-burden states, from the posterior samples of malaria mortality for the years 2004 and 2013 are presented in Fig. 3. The decline in the risk of malaria mortality over the years is evident across the high-burden states. A summary of the estimated model parameters is presented in Table 2.

**Table 2:** Parameter estimates (posterior medians) and 95% credible intervals of log Relative Risk and variance parameters from the spatio-temporal model

Model parameters	Estimate	95% CI^a^
Urban/Rural status (reference: Rural)	− 0.618	(− 0.735, 0.504)
NDVI	0.688	(0.100, 1.279)
Temporal standard deviation	0.003	(0.002, 0.007)
Spatio-temporal standard deviation	0.008	(0.004, 0.023)
Spatial–temporal Range	15.600	(3.680, 90.500)
Standard deviation for SRS units random effect	0.536	(0.481, 0.610)
Spatial standard deviation	1.101	(0.928, 1.355)
Spatial Range (km)	12.100	(9.5200, 16.100)
Spatio-temporal correlation	0.006	(− 0.250, 0.262)
Intercept	− 0.520	(− 1.131, 0.040)

^a^*CI* Credible interval, The 95% CIs are based on posterior 2.5th and 97.5th quantiles

**Fig. 4:**
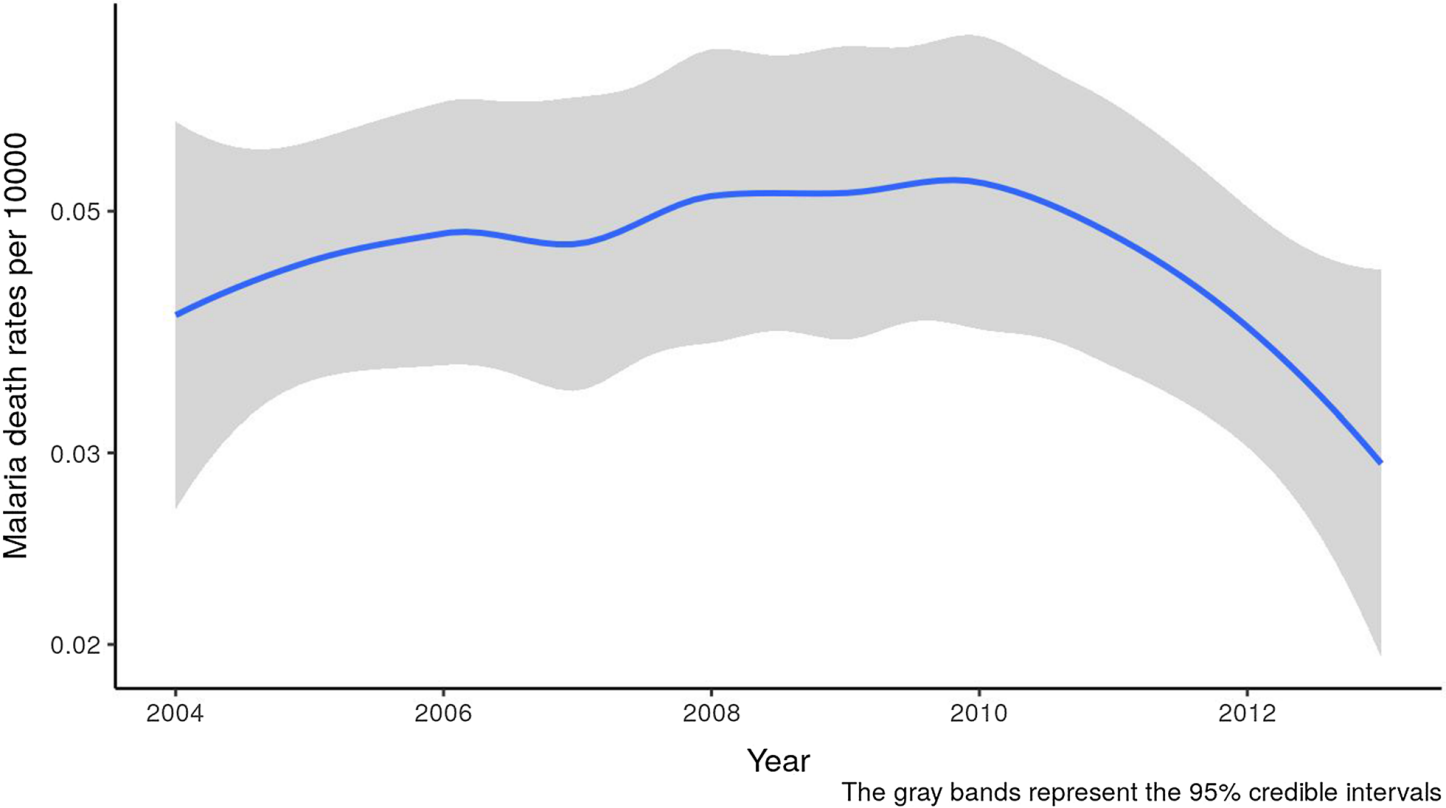
Temporal decline of malaria mortality across the country. Solid blue line represents temporal decline

Table 2 presents the parameter estimates and 95% credible intervals for the hyperparameters of the spatio-temporal models. The details of the model and its parameters are provided in Additional file 1: Appendix A.1. Compared to the rural population, the urban population is at a 1 − exp(− 0.618) * 100 = 47% decreased risk of dying of malaria. This is consistent with findings in the exploratory analysis, where it was observed that the rural population is more vulnerable than the urban population. The posterior quantiles, that is the 95% quantiles from the posterior distribution, of the correlation between the two spatial effects is shown in the rows marked “spatio-temporal correlation” in Table 2, and the correlation is not significantly different from zero. Figure 4 confirms an overall temporal decline in malaria mortality.

**Fig. 5:**
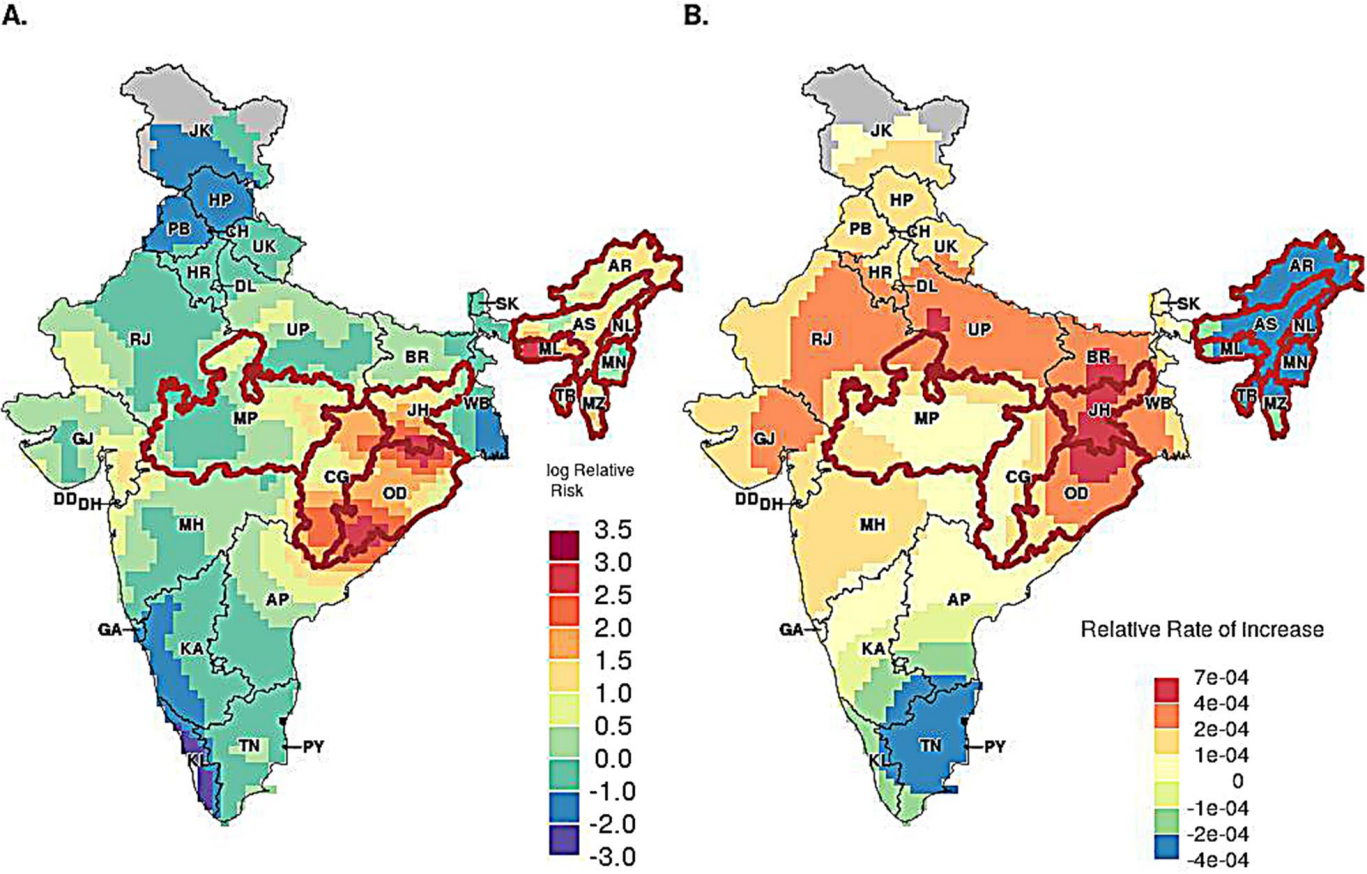
Posterior medians of spatial random effects. **A** Posterior medians of spatial random effects—log of relative risk of malaria mortality, **B** Posterior medians of spatial random effects—annual decline of malaria mortality. State boundaries are based on the 2011 census and hence exclude Telangana. The brown boundaries have been used to highlight the high-burden states. Data are unavailable for the grey shaded region. (State abbreviations: *JK* ‘Jammu & Kashmir', *HP* 'Himachal Pradesh', *PB* 'Punjab', *CH* 'Chandigarh', *UK* 'Uttarakhand', *HR* 'Haryana', *DL* 'Delhi', *RJ* 'Rajasthan', *UP* 'Uttar Pradesh', *BR* 'Bihar', *SK* 'Sikkim', *AP* 'Arunachal Pradesh', *NL* 'Nagaland', *MN* 'Manipur', *MZ* 'Mizoram', *TR* 'Tripura', *ML* 'Meghalaya', *AS* 'Assam', WB 'West Bengal', *JH* 'Jharkhand', *OD* 'Odisha', *CH* 'Chhattisgarh', *MP* 'Madhya Pradesh', *GJ* 'Gujarat', *DD* 'Daman & Diu', *DH* 'Dadra & Nagar Haveli', *MH* 'Maharashtra', *KA* 'Karnataka', *GA* 'Goa', *KL* 'Kerala', TN 'Tamil Nadu', *PY* 'Puducherry', *AP* 'Andhra Pradesh')

The spatial distribution of annual decline in malaria mortality rates are presented in Fig. 5. In Fig. 5B, the red and orange regions with positive log relative risk (RR) values indicate increased risk of malaria mortality in these regions. The blue and green regions with negative log RR indicate regions with lower risk of malaria mortality. Figure 5B illustrates the rate of change of mortality risks across the country from 2004- 2013. Positive rate of declining mortality (i.e., faster) is denoted in orange, and negative rate of mortality decline (i.e., slower) is denoted in blue. The rate of decline is fastest in regions with the highest burden: Odisha and Jharkhand. However, the rate of decline is one of the slowest in the north-eastern states, despite it being one of the higher-risk regions. Outside of the five high-burden states, the rate of decline is the slowest in southern states of Tamil Nadu and Kerala. The rate of decline in the northern states, especially in the states of Uttar Pradesh and Bihar, is at par with those in Odisha and Jharkhand.

## Discussion

Malaria mortality data from the Indian MDS for the years 2004–2013, were analysed, with spatio-temporal models to study trends in space and time, by sex, urban/rural setting, and NDVI. Malaria mortality data from the MDS for years 1998–2003, analysed by Dhingra et al. [2] included age distribution, spatial distribution, and proportion of home deaths. U-shaped patterns similar to Dhingra et al. [2] were observed in the high-burden states, and across all age-groups and sex-specific strata. In exploratory analysis, it was observed that the rural population had twice the risk of malaria mortality than urban dwellers. The difference is consistent geographically, by age group, and over time. Of note is that about a quarter of these deaths occurred in health facilities (Table 1).

Spatio-temporal studies have been helpful in directing malaria control strategies in other countries, e.g., a study in Bhutan indicated a need to prioritize high-risk clusters of the disease [29]; in Nepal, a spatio-temporal study on malaria led to the conclusion that preventive measures should be scaled up to entire districts and not just high endemicity areas [30]. All spatio-temporal studies have noted strong seasonal malaria patterns [4–6, 29, 30]. Other studies have claimed that climatic factors such as temperature, rainfall, humidity, vapour pressure, and wind velocity are significant predictors of spatio-temporal distribution of malaria [7, 31, 32]. Spatio-temporal studies in malaria from different countries have documented an overall declining trend, consistent with global statistics [33].

The spatio-temporal analysis in this study confirms the high-burden states as identified by earlier studies. The results highlight that the rate of decline in malaria mortality was fastest in the highest-burden states of Odisha and Jharkhand. However, for the north east states the rate of decline was one of the slowest. The improvement in Odisha might be attributed to employing auxiliary nurse midwives (ANMs) and accredited social health activist (ASHAs) to deliver artemisinin-combination therapy (ACT) and perform rapid diagnostic tests (RDTs), under the new malaria treatment policy of 2010 [34]. The spatio-temporal model estimates confirmed that the rural populations were at a higher risk of malaria mortality.

A central hypothesis of this work was that malaria mortality rates had declined fastest where malaria mortality was more prevalent. The spatio-temporal model found this not to be the case—in relative terms declines were uniform across India. In absolute terms declines were greater in the high-risk areas, as a 10% decline from a high baseline is a larger mortality reduction per unit of population than a comparable decline in a low-risk area.

Malaria mortality data from the MDS are consistent with data from the NVBDCP [35] in identifying high-burden states (Additional file 1: Appendix A.3). A common feature of these states is the dominance of tribal populations, which are often less accessible, and lack adequate infrastructure and disease surveillance compared to other areas. Other studies have also observed tribal populations to be more vulnerable to the disease [36]. Ahmad et al. [3] have provided evidence of malaria-affected regions in Jharkhand, which has a tribal-dominant population. In the current study, tribal versus non-tribal regions have not been compared, but this could be done in the future. Future studies could also compare the variation in spatio-temporal distribution between children and adults. Earlier studies from different states of India present contradictory results on the comparison of malaria incidence among children and adults [15, 37–39]. For example, in the state of Rajasthan and in some of the north-eastern states, namely Assam and Arunachal Pradesh, children had a higher incidence of malaria, whereas it was an entirely opposite scenario in the indo-gangetic plains, where adults were more vulnerable than children [40].

This study has some limitations. One is the reliance of the MDS on VA, which have some potential biases, because of a lack of medically-certified causes of deaths. There are both pros and cons of VA methods [24]. Misclassification of acute febrile deaths by VA among medically unattended adults is not unexpected, and likely results in malaria being over- diagnosed in some cases and under-diagnosed in others [1, 2]. Since this study relied only on mortality data, and comparable incidence data were not available, therefore case fatality ratios across space and time could not be compared. Another limitation is that the current INLA model for the spatio-temporal analyses of malaria mortality does not accommodate analysing seasonality. One future direction of research could be adding seasonality for spatio-temporal modelling in the INLA. Finally, progress has been made in malaria control in some areas over the past few years, so this analysis should be updated as new data become available, and new interventions and policies should reflect current conditions, to the extent possible.

## Conclusion

The analysis and models presented in this paper assessed spatial and temporal dynamics of malaria mortality across all age groups and for males and females, in India, from 2004 to 2013, using death records from a decade-long household mortality survey — the Million Death Study. Some spatial and temporal variations were observed across the country. Spatial variations vary across the time period, but there is an overall declining trend across the country. The high-burden states identified by earlier studies were confirmed as high-risk regions in this study. Lack of spatio-temporal correlation established that there is no association between the temporal trends and risk of malaria mortality in a region.

These results suggest that control programmes and strategies with greater emphasis may be required in some parts of the country. The analyses presented in this paper should help state and national malaria control efforts better target high-risk areas and populations, towards the goal of eventual malaria elimination from India. Collection and release of more current data will be needed to address the situation appropriately, however.

## Supplementary Information

**Additional file 1.** Appendices.

## Data Availability

Under legal agreement with the Registrar General of India, the MDS data cannot be redistributed outside of the Centre for Global Health Research. Please refer to http://www.censusindia.gov.in/vital_statistics/SRS_Statistical_Report.html for public reports. For MDS data access procedures, please contact the Office of the Registrar General, RK Puram, New Delhi, India (rgoffice.rgi@nic.in). Sample R codes for statistical analysis can be obtained from the first author.

## Abbreviations

MDS: Million Deaths Study
RGI: Registrar General of India
COD: Cause of death
SRS: Sample Registration System
CGHR: Centre for Global Health Research
VA: Verbal Autopsy
ICD-10: International Statistical Classification of Diseases and Related Health Problems 10th Revision
WHO: World Health Organization
UN: United Nations
GLGM: Generalized Linear Geostatistical Models
INLA: Integrated Nested Laplace Approximation
RR: Relative Risk
CI: Credible Interval
