# Supplementary material for: Spatio-temporal modelling of malaria mortality in India from 2004 to 2013 from the Million Death Study
Source: Malar J. 2022 Mar 17;21:90. doi: 10.1186/s12936-022-04112-x (PMC8932160; doi:10.1186/s12936-022-04112-x)
Supplement: Supplementary file 1 — Additional file 1. Appendices. [file 12936_2022_4112_MOESM1_ESM.docx]

**Appendix A.1**

Suppose, $y_{it}$ is the outcome recorded at sampling unit $i$ (located at the spatial coordinates $s_{i}$) and time $t$. The expected number of malaria deaths, $E_{it}$ are obtained from age-specific population counts and baseline rates computed using a simple non-spatial model with age and time terms. The log of relative rate is linearly modelled on unit-level covariates $X_{1i}$, which in this study includes only the urban/rural indicator variable, $X_{2i}$ which includes NDVI as a covariate, $\alpha$ and $\beta$ are the coefficient, and $b_{0}$ is an intercept. The $\gamma_{t}$ is a temporal trend and $Z_{i}$ is the sampling unit-level random effect. The sampling unit-level random effect are spatially independent and accounts for short-scale spatial variance or sampling unit-level risk factors which are otherwise not included as model covariates. The model is written as

$$y_{it}\sim Poisson\left( E_{it}\rho_{it} \right)$$

$$log\left( \rho_{it} \right)=b_{0}+X_{1i}\alpha+X_{2i}\beta+ U_{1}\left( s_{i} \right)+tU_{2}\left( s_{i} \right)+Z_{i}+\gamma_{t},$$

The spatial random effect $U_{1}\left( s \right)$ is a smoothly-varying surface which represents possible spatially varying risk factors. A second spatial effect $U_{2}\left( s \right)$ is a ‘random slope’ for time, to estimate if malaria mortality is decreasing more quickly where $U_{2}\left( s \right)$ is small than in locations where $U_{2}\left( s \right)$ is large. More specifically, the spatial effects are independent stationary Gaussian random field with a Matérn [1] correlation function $\omega$ with

$$Cov\left( U_{k}\left( s+h \right),U_{k}\left( s \right) \right)=\sigma^{2}\omega_{k}\left( s,\phi\right),$$

where the range parameter $\phi$ determines the smoothness of the surface, *h* stands for any amount of distance, *k =*1, 2.

The temporal random effect $\gamma_{t}$ is dynamically modelled as a Random Walk (RW) model of order 2. Here *t* is the time effect in 10-year increments, centred at the year 2010. Therefore, *t* is in the range of -0.6 to 0.4. RW models introduced by Karl Pearson [2] and solved by Rayleigh [3], have a wide range of applications, ranging from ecology, economics and spatial statistics. A RW(2) has been used to model the temporal effect. The prime purpose of using a RW model was to achieve “smooth effects” and an RW(2) model was used for even “smoother effects” than could be achieved by the RW(1) model.

The prior for the standard deviation parameters of each of the random effects, in all the models considered, are exponential priors. These priors assume that the variance is close to 0, so that the model has fewer fluctuations, and the data must provide information on the spatial distribution of risk in order for the surfaces to have higher- and lower-valued areas.

**Appendix A.2**
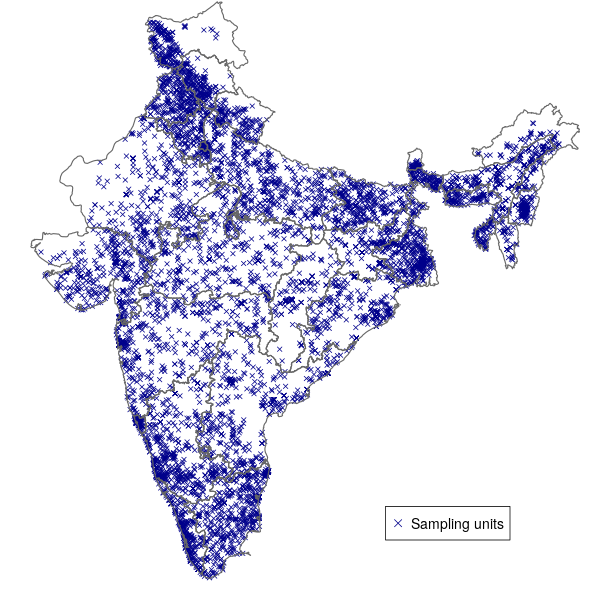


Figure 1: Distribution of the SRS units covered in MDS 2004-13

**Appendix A.3**

Table 1: Comparison of MDS and NVBDCP malaria mortality counts and percentage distribution, 2004-13

| States | 2004-06 | | | | 2007-10 | | | | 2011-13 | | | |
| --- | --- | --- | --- | --- | --- | --- | --- | --- | --- | --- | --- | --- |
|  | MDS^2^ | | NVBDCP^3^ | | MDS | | NVBDCP | | MDS | | NVBDCP | |
|  | Counts | %^1^ | counts | %^1^ | counts | %^1^ | counts | %^1^ | counts | %^1^ | counts | %^1^ |
| Odisha | 710 | 21 | 795 | 22 | 1054 | 32 | 905 | 20 | 630 | 24 | 245 | 14 |
| NE | 645 | 19 | 1332 | 37 | 925 | 28 | 1707 | 38 | 417 | 16 | 394 | 23 |
| MP | 210 | 6 | 136 | 4 | 249 | 8 | 151 | 3 | 97 | 4 | 201 | 12 |
| Chhattisgarh | 97 | 3 | 10 | 0 | 323 | 10 | 62 | 1 | 65 | 3 | 175 | 10 |
| Jharkhand | 78 | 2 | 65 | 2 | 109 | 3 | 100 | 2 | 86 | 3 | 35 | 2 |
| Other states & UT | 1573 | 47 | 1281 | 35 | 624 | 19 | 1608 | 35 | 1300 | 50 | 663 | 39 |
| India | 3313 | 100 | 3619 | 100 | 3284 | 100 | 4533 | 100 | 2595 | 100 | 1713 | 100 |

Totals may not equal 100% due to rounding.

^2^ Death assigned by VA.

^3^ NVBDCP slide-positive clinically confirmed malaria deaths.

The MDS malaria mortality data are broadly consistent with microbiologically-confirmed deaths (via slide testing or rapid diagnostic tests) among febrile patients at primary health care centres collected by the NVBDCP in the high-burden states. Despite the differences in method of death counting by both surveys, resulting in modest variations in the counts and percentages, a generally decreasing trend in the recent years has been captured by both surveys. Similar and more detailed comparisons of the results from MDS and NVBDCP can be found in Dhingra et al., [4] and Cohen et al., [5].

**Appendix A.4**

Table 4.1: Malaria-attributed deaths, from MDS Round 2 for Odisha, by age

| **2004-06** | | | | | | | |
| --- | --- | --- | --- | --- | --- | --- | --- |
| **Age Group** | **Numbers attributed to malaria deaths** | **All coded deaths** | **Number of deaths attributed to malaria/all coded deaths** | **Died in a health facility** | **Proportion died in a health facility** | **Rural** | **Proportion from rural area** |
| 1-59 months | 116 | 737 | 15.7 | 26 | 22.4 | 109 | 94 |
| 5-14 years | 75 | 292 | 25.7 | 25 | 33.3 | 69 | 92 |
| 15-29 years | 90 | 558 | 16.1 | 31 | 34.4 | 81 | 90 |
| 30-44 years | 73 | 635 | 11.5 | 24 | 32.9 | 62 | 84.9 |
| 45-59 years | 112 | 999 | 11.2 | 17 | 15.2 | 100 | 89.3 |
| 60-69 years | 131 | 1310 | 10 | 13 | 9.9 | 124 | 94.7 |
| Summary (0-69 years) | 597 | 4531 | 13.2 | 136 | 22.8 | 545 | 91.3 |
| >70 years | 111 | 2238 | 5 | 9 | 8.1 | 102 | 91.9 |
| **2007-10** | | | | | | | |
| 1-59 months | 130 | 785 | 16.6 | 39 | 30 | 125 | 96.2 |
| 5-14 years | 84 | 331 | 25.4 | 30 | 35.7 | 75 | 89.3 |
| 15-29 years | 125 | 756 | 16.5 | 49 | 39.2 | 111 | 88.8 |
| 30-44 years | 108 | 989 | 10.9 | 26 | 24.1 | 94 | 87 |
| 45-59 years | 189 | 1547 | 12.2 | 30 | 15.9 | 170 | 89.9 |
| 60-69 years | 182 | 1925 | 9.5 | 20 | 11 | 159 | 87.4 |
| Summary  (0-69 years) | 818 | 6333 | 12.9 | 194 | 23.7 | 734 | 89.7 |
| >70 years | 235 | 3473 | 6.8 | 17 | 7.2 | 210 | 89.4 |
| **2011-13** | | | | | | | |
| **Age Group** | **Numbers attributed to malaria deaths** | **All coded deaths** | **Numbers of attributed to malaria/all coded deaths** | **Died in a health facility** | **Proportion died in a health facility** | **Rural** | **Proportion from rural area** |
| 1-59 months | 49 | 335 | 14.6 | 11 | 22.4 | 44 | 89.8 |
| 5-14 years | 28 | 170 | 16.5 | 13 | 46.4 | 26 | 92.9 |
| 15-29 years | 77 | 533 | 14.4 | 28 | 36.4 | 72 | 93.5 |
| 30-44 years | 87 | 727 | 12 | 28 | 32.2 | 77 | 88.5 |
| 45-59 years | 125 | 1281 | 9.8 | 22 | 17.6 | 110 | 88 |
| 60-69 years | 127 | 1616 | 7.9 | 12 | 9.4 | 107 | 84.3 |
| Summary (0-69 years) | 493 | 4662 | 10.6 | 114 | 23.1 | 436 | 88.4 |
| >70 years | 134 | 2951 | 4.5 | 7 | 5.2 | 122 | 91 |

Table 4.2: Malaria-attributed deaths, from MDS Round 2 for the North-Eastern States combined, by age

| **2004-06** | | | | | | | |
| --- | --- | --- | --- | --- | --- | --- | --- |
| **Age Group** | **Numbers attributed to malaria deaths** | **All coded deaths** | **Numbers of attributed to malaria/all coded deaths** | **Died in a health facility** | **Proportion died in a health facility** | **Rural** | **Proportion from rural area** |
| 1-59 months | 138 | 967 | 14.3 | 28 | 20.3 | 115 | 83.3 |
| 5-14 years | 128 | 490 | 26.1 | 23 | 18 | 107 | 83.6 |
| 15-29 years | 114 | 766 | 14.9 | 39 | 34.2 | 89 | 78.1 |
| 30-44 years | 90 | 1054 | 8.5 | 32 | 35.6 | 68 | 75.6 |
| 45-59 years | 73 | 1398 | 5.2 | 14 | 19.2 | 53 | 72.6 |
| 60-69 years | 33 | 1238 | 2.7 | 8 | 24.2 | 28 | 84.8 |
| Summary (0-69 years) | 576 | 5913 | 9.7 | 144 | 25 | 460 | 79.9 |
| >70 years | 66 | 2372 | 2.8 | 14 | 21.2 | 52 | 78.8 |
| **2007-10** | | | | | | | |
| **Age Group** | **Numbers attributed to malaria deaths** | **All coded deaths** | **Numbers of attributed to malaria/all coded deaths** | **Died in a health facility** | **Proportion died in a health facility** | **Rural** | **Proportion from rural area** |
| 1-59 months | 130 | 785 | 16.6 | 39 | 30 | 125 | 96.2 |
| 5-14 years | 84 | 331 | 25.4 | 30 | 35.7 | 75 | 89.3 |
| 15-29 years | 125 | 756 | 16.5 | 49 | 39.2 | 111 | 88.8 |
| 30-44 years | 108 | 989 | 10.9 | 26 | 24.1 | 94 | 87 |
| 45-59 years | 189 | 1547 | 12.2 | 30 | 15.9 | 170 | 89.9 |
| 60-69 years | 182 | 1925 | 9.5 | 20 | 11 | 159 | 87.4 |
| Summary (0-69 years) | 818 | 6333 | 12.9 | 194 | 23.7 | 734 | 89.7 |
| >70 years | 235 | 3473 | 6.8 | 17 | 7.2 | 210 | 89.4 |
| **2011-13** | | | | | | | |
| **Age Group** | **Numbers attributed to malaria deaths** | **All coded deaths** | **Numbers of attributed to malaria/all coded deaths** | **Died in a health facility** | **Proportion died in a health facility** | **Rural** | **Proportion from rural area** |
| 1-59 months | 49 | 335 | 14.6 | 11 | 22.4 | 44 | 89.8 |
| 5-14 years | 28 | 170 | 16.5 | 13 | 46.4 | 26 | 92.9 |
| 15-29 years | 77 | 533 | 14.4 | 28 | 36.4 | 72 | 93.5 |
| 30-44 years | 87 | 727 | 12 | 28 | 32.2 | 77 | 88.5 |
| 45-59 years | 125 | 1281 | 9.8 | 22 | 17.6 | 110 | 88 |
| 60-69 years | 127 | 1616 | 7.9 | 12 | 9.4 | 107 | 84.3 |
| Summary (0-69 years) | 493 | 4662 | 10.6 | 114 | 23.1 | 436 | 88.4 |
| >70 years | 134 | 2951 | 4.5 | 7 | 5.2 | 122 | 91 |

Table 4.3: Malaria-attributed deaths, from MDS Round 2 for Chhattisgarh, by age

| **2004-06** | | | | | | | |
| --- | --- | --- | --- | --- | --- | --- | --- |
| **Age Group** | **Numbers attributed to malaria deaths** | **All coded deaths** | **Numbers of attributed to malaria/all coded deaths** | **Died in a health facility** | **Proportion died in a health facility** | **Rural** | **Proportion from rural area** |
| 1-59 months | 19 | 152 | 12.5 | 0 | 0 | 18 | 94.7 |
| 5-14 years | 10 | 74 | 13.5 | 1 | 10 | 9 | 90 |
| 15-29 years | 5 | 128 | 3.9 | 1 | 20 | 5 | 100 |
| 30-44 years | 11 | 209 | 5.3 | 5 | 45.5 | 8 | 72.7 |
| 45-59 years | 19 | 280 | 6.8 | 2 | 10.5 | 11 | 57.9 |
| 60-69 years | 18 | 367 | 4.9 | 1 | 5.6 | 13 | 72.2 |
| Summary (0-69 years) | 82 | 1210 | 6.8 | 10 | 12.2 | 64 | 78.0 |
| >70 years | 15 | 470 | 3.2 | 0 | 0 | 8 | 53.3 |
| **2007-10** | | | | | | | |
| **Age Group** | **Numbers attributed to malaria deaths** | **All coded deaths** | **Numbers of attributed to malaria/all coded deaths** | **Died in a health facility** | **Proportion died in a health facility** | **Rural** | **Proportion from rural area** |
| 1-59 months | 12 | 187 | 6.4 | 2 | 16.7 | 11 | 91.7 |
| 5-14 years | 11 | 88 | 12.5 | 5 | 45.5 | 7 | 63.6 |
| 15-29 years | 14 | 232 | 6 | 9 | 64.3 | 7 | 50 |
| 30-44 years | 19 | 328 | 5.8 | 4 | 21.1 | 14 | 73.7 |
| 45-59 years | 19 | 504 | 3.8 | 4 | 21.1 | 11 | 57.9 |
| 60-69 years | 13 | 580 | 2.2 | 1 | 7.7 | 11 | 84.6 |
| Summary (0-69 years) | 88 | 1919 | 4.6 | 25 | 28.4 | 61 | 69.3 |
| >70 years | 235 | 3473 | 6.8 | 17 | 7.2 | 210 | 89.4 |
| **2011-13** | | | | | | | |
| **Age Group** | **Numbers attributed to malaria deaths** | **All coded deaths** | **Numbers of attributed to malaria/all coded deaths** | **Died in a health facility** | **Proportion died in a health facility** | **Rural** | **Proportion from rural area** |
| 1-59 months | 6 | 120 | 5 | 1 | 16.7 | 3 | 50 |
| 5-14 years | 2 | 46 | 4.3 | 1 | 50 | 2 | 100 |
| 15-29 years | 3 | 196 | 1.5 | 1 | 33.3 | 0 | 0 |
| 30-44 years | 9 | 252 | 3.6 | 3 | 33.3 | 3 | 33.3 |
| 45-59 years | 14 | 413 | 3.4 | 6 | 42.9 | 8 | 57.1 |
| 60-69 years | 10 | 405 | 2.5 | 2 | 20 | 9 | 90 |
| Summary (0-69 years) | 44 | 1432 | 3.1 | 14 | 31.9 | 25 | 56.9 |
| >70 years | 20 | 633 | 3.2 | 0 | 0 | 8 | 40 |

Table 4.4: Malaria-attributed deaths, from MDS Round 2 for Madhya Pradesh, by age

| **2004-06** | | | | | | | |
| --- | --- | --- | --- | --- | --- | --- | --- |
| **Age Group** | **Numbers attributed to malaria deaths** | **All coded deaths** | **Numbers of attributed to malaria/all coded deaths** | **Died in a health facility** | **Proportion died in a health facility** | **Rural** | **Proportion from rural area** |
| 1-59 months | 59 | 975 | 6.1 | 3 | 5.1 | 53 | 89.8 |
| 5-14 years | 29 | 328 | 8.8 | 4 | 13.8 | 28 | 96.6 |
| 15-29 years | 26 | 474 | 5.5 | 7 | 26.9 | 21 | 80.8 |
| 30-44 years | 15 | 519 | 2.9 | 2 | 13.3 | 11 | 73.3 |
| 45-59 years | 22 | 726 | 3 | 2 | 9.1 | 19 | 86.4 |
| 60-69 years | 26 | 897 | 2.9 | 0 | 0 | 22 | 84.6 |
| Summary (0-69 years) | 177 | 3919 | 4.5 | 18 | 10.2 | 154 | 87.0 |
| >70 years | 33 | 1602 | 2.1 | 1 | 3 | 31 | 93.9 |
| **2008-10** | | | | | | | |
| **Age Group** | **Numbers attributed to malaria deaths** | **All coded deaths** | **Numbers of attributed to malaria/all coded deaths** | **Died in a health facility** | **Proportion died in a health facility** | **Rural** | **Proportion from rural area** |
| 1-59 months | 60 | 1103 | 5.4 | 15 | 25 | 53 | 88.3 |
| 5-14 years | 29 | 350 | 8.3 | 4 | 13.8 | 27 | 93.1 |
| 15-29 years | 29 | 713 | 4.1 | 7 | 24.1 | 26 | 89.7 |
| 30-44 years | 18 | 739 | 2.4 | 5 | 27.8 | 15 | 83.3 |
| 45-59 years | 22 | 1067 | 2.1 | 3 | 13.6 | 19 | 86.4 |
| 60-69 years | 34 | 1378 | 2.5 | 2 | 5.9 | 32 | 94.1 |
| Summary (0-69 years) | 192 | 5350 | 3.6 | 36 | 18.7 | 172 | 89.6 |
| >70 years | 56 | 2301 | 2.4 | 4 | 7.1 | 47 | 83.9 |
| **2011-13** | | | | | | | |
| **Age Group** | **Numbers attributed to malaria deaths** | **All coded deaths** | **Numbers of attributed to malaria/all coded deaths** | **Died in a health facility** | **Proportion died in a health facility** | **Rural** | **Proportion from rural area** |
| 1-59 months | 18 | 523 | 3.4 | 6 | 33.3 | 14 | 77.8 |
| 5-14 years | 6 | 500 | 1.2 | 1 | 16.7 | 6 | 100 |
| 15-29 years | 13 | 581 | 2.2 | 6 | 46.2 | 10 | 76.9 |
| 30-44 years | 5 | 580 | 0.9 | 1 | 20 | 4 | 80 |
| 45-59 years | 23 | 870 | 2.6 | 8 | 34.8 | 17 | 73.9 |
| 60-69 years | 15 | 1025 | 1.5 | 4 | 26.7 | 14 | 93.3 |
| Summary (0-69 years) | 80 | 4079 | 2.0 | 26 | 32.5 | 65 | 81.2 |
| >70 years | 16 | 2033 | 0.8 | 0 | 0 | 13 | 81.3 |

Table 4.5: Malaria-attributed deaths, from MDS Round 2 for Jharkhand, by age

| **2004-06** | | | | | | | |
| --- | --- | --- | --- | --- | --- | --- | --- |
| **Age Group** | **Numbers attributed to malaria deaths** | **All coded deaths** | **Numbers of attributed to malaria/all coded deaths** | **Died in a health facility** | **Proportion died in a health facility** | **Rural** | **Proportion from rural area** |
| 1-59 months | 12 | 283 | 4.2 | 3 | 25 | 9 | 75 |
| 5-14 years | 8 | 118 | 6.8 | 0 | 0 | 7 | 87.5 |
| 15-29 years | 8 | 155 | 5.2 | 3 | 37.5 | 5 | 62.5 |
| 30-44 years | 14 | 212 | 6.6 | 4 | 28.6 | 11 | 78.6 |
| 45-59 years | 17 | 328 | 5.2 | 3 | 17.6 | 12 | 70.6 |
| 60-69 years | 8 | 403 | 2 | 1 | 12.5 | 4 | 50 |
| Summary (0-69 years) | 67 | 1499 | 4.5 | 14 | 20.9 | 48 | 71.6 |
| >70 years | 11 | 501 | 2.2 | 1 | 9.1 | 5 | 45.5 |
| **2007-10** | | | | | | | |
| **Age Group** | **Numbers attributed to malaria deaths** | **All coded deaths** | **Numbers of attributed to malaria/all coded deaths** | **Died in a health facility** | **Proportion died in a health facility** | **Rural** | **Proportion from rural area** |
| 1-59 months | 18 | 247 | 7.3 | 5 | 27.8 | 12 | 66.7 |
| 5-14 years | 10 | 121 | 8.3 | 0 | 0 | 6 | 60.0 |
| 15-29 years | 12 | 193 | 6.2 | 2 | 16.7 | 9 | 75 |
| 30-44 years | 17 | 281 | 6 | 4 | 23.5 | 13 | 76.5 |
| 45-59 years | 26 | 437 | 5.9 | 5 | 19.2 | 17 | 65.4 |
| 60-69 years | 13 | 473 | 2.7 | 1 | 7.7 | 11 | 84.6 |
| Summary (0-69 years) | 96 | 1752 | 5.5 | 17 | 17.7 | 68 | 70.8 |
| >70 years | 13 | 661 | 2 | 2 | 15.4 | 7 | 53.8 |
| **2011-13** | | | | | | | |
| **Age Group** | **Numbers attributed to malaria deaths** | **All coded deaths** | **Numbers of attributed to malaria/all coded deaths** | **Died in a health facility** | **Proportion died in a health facility** | **Rural** | **Proportion from rural area** |
| 1-59 months | 10 | 121 | 8.3 | 2 | 20 | 5 | 50 |
| 5-14 years | 7 | 69 | 10.1 | 0 | 0 | 7 | 100 |
| 15-29 years | 7 | 149 | 4.7 | 2 | 28.6 | 3 | 42.9 |
| 30-44 years | 16 | 202 | 7.9 | 3 | 18.8 | 10 | 62.5 |
| 45-59 years | 17 | 310 | 5.5 | 2 | 11.8 | 10 | 58.8 |
| 60-69 years | 15 | 375 | 4 | 2 | 13.3 | 9 | 60 |
| Summary (0-69 years) | 72 | 1226 | 5.9 | 11 | 15.3 | 44 | 61.1 |
| >70 years | 13 | 536 | 2.4 | 1 | 7.7 | 7 | 53.8 |

References

1. Matérn B. Spatial Variation. Springer-Verlag. New York. 1986.
2. Pearson K. The problem of the random walk. Nat. 1905;72:342.
3. Rayleigh L. The problem of the random walk. Nat. 1905;72:318.
4. Dhingra N, Jha P, Sharma VP, Cohen AA, Jotkar RM, Rodriguez PS, et al. Adult and child malaria mortality in India: a nationally representative mortality survey Lancet. 2010;376:1768-74.
5. Cohen AA, Dhingra N, Jotkar RM, Rodriguez PS, Sharma VP, Jha P. The Summary Index of Malaria Surveillance (SIMS): a stable index of malaria within India. Popul Health Metr. 2010;8:1.
